# RETRACTED: Paucarmayta et al. Progesterone-Calcitriol Combination Enhanced Cytotoxicity of Cisplatin in Ovarian and Endometrial Cancer Cells In Vitro. *Biomedicines* 2020, *8*, 73

**DOI:** 10.3390/biomedicines13071591

**Published:** 2025-06-30

**Authors:** Ana Paucarmayta, Hannah Taitz, Latoya McGlorthan, Yovanni Casablanca, G. Larry Maxwell, Kathleen M. Darcy, Viqar Syed

**Affiliations:** 1 Department of Obstetrics & Gynecology, Uniformed Services University, 4301 Jones Bridge Road, Bethesda, MD 20814, USA; ana.paucarmayta@gmail.com (A.P.); hptaitz@gmail.com (H.T.); latoya.mcglorthan.ctr@usuhs.edu (L.M.); yovanni.casablanca.mil@mail.mil (Y.C.); darcyk@whirc.org (K.M.D.); 2 Gynecologic Cancer Center of Excellence, Department of Obstetrics and Gynecology, Uniformed Services University of the Health Sciences and Walter Reed National Military Medical Center, 8901 Wisconsin Avenue, Bethesda, MD 20889, USA; 3 John P. Murtha Cancer Center, Uniformed Services University of the Health Sciences and Walter Reed National Military Medical Center, 8901 Wisconsin Avenue, Bethesda, MD 20889, USA; george.maxwell@inova.org; 4 Gynecologic Cancer Center of Excellence, Women’s Health Integrated Research Center at Inova Health System, 3289 Woodburn Road, Suite 370, Annandale, VA 22003, USA; 5 Inova Fairfax Hospital, Department of Obstetrics & Gynecology, 3300 Gallows Road, Falls Church, VA 22042, USA; 6 Department of Molecular and Cell Biology, Uniformed Services University, 4301 Jones Bridge Road, Bethesda, MD 20814, USA

The journal retracts the article, “Progesterone-Calcitriol Combination Enhanced Cytotoxicity of Cisplatin in Ovarian and Endometrial Cancer Cells In Vitro” [1], cited above.

Following publication, concerns were brought to the attention of the publisher regarding image duplication and the overall reliability of several figures presented in this article.

Adhering to our standard procedure, an investigation was conducted by the Editorial Office and Editorial Board, with input from the Uniformed Services University of the Health Sciences, USA. The investigation confirmed duplications between Figure 4A, ERK1/2 panel [1] and Figure 5D, ERK panel published in [2] and between Figure 5C, ABCG1 panel [1] and Figure 3C, p-STAT 3 panel [3], presenting different experimental conditions and originating from a different authorship group. As a result, the Editorial Board has lost confidence in the integrity of the findings and has decided to retract this article [1] as per MDPI’s retraction policy (https://www.mdpi.com/ethics#_bookmark30, accessed on 12 March 2025).

This retraction was approved by the Editor-in-Chief of the journal *Biomedicines*.

A.P., H.T., L.M., Y.C., G.L.M., and K.M.D. agreed to the retraction. The remaining author did not provide a comment on this decision.
